# Muscle loss in cancer cachexia: what is the basis for nutritional support?

**DOI:** 10.3389/fphar.2025.1519278

**Published:** 2025-02-26

**Authors:** Jaline Faiad, Márcia Fábia Andrade, Gabriela de Castro, Joyce de Resende, Marina Coêlho, Giovana Aquino, Marilia Seelaender

**Affiliations:** Cancer Metabolism Research Group, Faculdade de Medicina da Universidade de São Paulo, Departamento de Cirurgia, LIM 26-HC-USP, São Paulo, Brazil

**Keywords:** nutritional supplementation, cancer cachexia, muscle wasting, chronic inflammation, protein synthesis

## Abstract

Cancer cachexia (CC) is characterized by significant skeletal muscle wasting, and contributes to diminished quality of life, while being associated with poorer response to treatment and with reduced survival. Chronic inflammation plays a central role in driving CC progression, within a complex interplay favoring catabolism. Although cachexia cannot be fully reversed by conventional nutritional support, nutritional intervention shows promise for the prevention and treatment of the syndrome. Of special interest are nutrients with antioxidant and anti-inflammatory potential and those that activate pathways involved in muscle mass synthesis and/or in the inhibition of muscle wasting. Extensive research has been carried out on novel nutritional supplements’ power to mitigate CC impact, while the mechanisms through which some nutrients or bioactive compounds exert beneficial effects on muscle mass are still not totally clear. Here, we discuss the most studied supplements and nutritional strategies for dealing with muscle loss in CC.

## 1 Introduction

Cancer cachexia (CC) is characterized by progressive functional debilitation and significant skeletal muscle mass loss, often accompanied by fat mass wasting. The decrease in muscle mass contributes to diminished quality of life, increased fatigue and morbidity, and is associated with poorer responses to oncological therapy (von Haehling and Anker, 2014). Cachexia related muscle loss frequently provokes, the early discontinuation of treatment, the necessity of chemotherapeutic drug dose adjustment, and is robustly linked with worsened prognosis and decreased survival (Mattox, 2017).

Cancer cachexia definition and staging are still controversial, which makes it difficult to compare the existing data and to determine its prevalence (Wiegert et al., 2020). Within the most widely accepted consensus (Fearon et al., 2011), the syndrome is divided into three progressive stages based on clinical and biochemical parameters: pre-cachexia, cachexia, and refractory cachexia (RCa), the latter being characterized by non-responsiveness to anticancer therapies and an expected survival of up to 3 months, solely (Fearon et al., 2011). Wiegert et al. (2020) compared three diagnostic criteria for cachexia in 1,384 patients with incurable cancer under palliative care. According to their findings, 17.3% of the patients were cachectic, 20.8% were in pre-cachexia, 53.3% were in the RCa stage, and 8.2% were non-cachectic by Viagno et al.‘s classification. Applying Blum et al.'s criteria, 53.9% of the patients were classified as cachectic, 12.3% as pre-cachectic, 26.1 as RCa, and 9.7% as non-cachectic. In contrast, Wallengren et al.'s criteria identified 13.8% of the patients as cachectic and 86.2% as non-cachectic. Cachexia was found to be most prevalent among patients with gastrointestinal tract tumors (Wiegert et al., 2020). On the other hand, a study by Orellana López et al. (2023), which determined cachexia prevalence using the miniCASCO tool in a cohort of cancer patients in Chile, found that 27.5% presented cachexia. Within this group, 45.45% could be classified to be in the stage of pre-cachexia, and 36.36% in RCa, and 18.18%, cachexia. Both studies underscore the importance of CC classification to clinical practice and its potential to guide treatment decisions effectively, particularly at the early stages of the syndrome (Wiegert et al., 2020; Orellana López et al., 2023).

Despite the traditional definition that cachexia cannot be fully reversed by conventional nutritional support (Fearon et al., 2011), multimodal interventions at the onset of the syndrome appear to allow some gains. An anabolic window is observed in patients with cancer cachexia (CC), particularly at the earlier stages of the disease or during periods of clinical stability. These phases are characterized by improved symptom control, optimized pain management, enhanced nutritional intake, and better physical performance (Prado et al., 2013). Later along the progression of the disease, many factors lead to possible anabolic resistance, such as tumor site and stage, anticancer treatment side-effects, life expectancy, nutritional status and dietary intake, and inflammation (Engelen et al., 2016).

The difficulties in cachexia management arise from the fact that the disease is *per se*, multifactorial in nature, and is driven by a combination of inflammation, disrupted metabolic processes, and negative energy and protein balance (Fearon et al., 2011). Chronic inflammation plays a central role in CC onset and progression, promoting tumor-host interaction mediated by pro-inflammatory cytokines (Donohoe et al., 2011). Tumor Necrosis Factor-alpha (TNF-α), also known as cachectin, is one of the key cytokines involved in CC (Fearon et al., 2012). It promotes protein degradation by activating the intracellular NF-κB pathway, which induces the expression of genes associated with the process of proteolysis, including Muscle RING-Finger protein-1 (MuRF1) and atrogin-1 (Armstrong et al., 2020). Interleukin (IL)-6 is another crucial cytokine in cachexia (Fearon et al., 2012). It activates STAT3, which then translocates to the nucleus, promoting the expression of genes involved in protein degradation and inflammation, hence exacerbating tissue loss (Martin et al., 2023; Fonseca et al., 2020). Furthermore, myostatin and activin A, members of the TGF-β superfamily, act as negative regulators of muscle mass by activating the SMAD2/3 pathway, which represses muscle differentiation and regeneration (Fearon et al., 2012). FoxO transcription factors also contribute to muscle atrophy, by up regulating ubiquitin ligases and promoting autophagy (Martin et al., 2023). TWEAK, another cytokine reported to be augmented in CC, exacerbates muscle wasting by inducing proteolytic enzymes and promoting inflammation (Armstrong et al., 2020; Martin et al., 2023). Another highly relevant catabolic factor associated with CC is Growth Differentiation Factor 15 (GDF15), a cytokine released in response to several stress signals. Augmented circulating levels of GDF15 may lead to weight loss and anorexia in patients with CC and are associated with reduced survival (Lerner et al., 2015; Suzuki et al., 2021). To our knowledge, no published studies have established a correlation among nutritional supplementation, GDF15 levels, and muscle mass regulation.

The pathophysiology of CC involves a complex interplay between anabolic and catabolic pathways, with a pronounced shift towards protein degradation in detriment of synthesis. In anabolic states, protein synthesis exceeds degradation resulting in muscle protein gain, while the opposite leads to catabolism, resulting in muscle mass loss (Stipanuk, 2008). The Mammalian Target of the Rapamycin (mTOR) pathway, regulated through the IGF-1/PI3K/AKT cascade, has a central role in promoting muscle protein synthesis (Yoshida and Delafontaine, 2020). However, in CC, mTOR signaling is often inhibited due to increased levels of proinflammatory cytokines, such as TNF-α (Fonseca et al., 2020).

Muscle loss in CC is predominantly mediated by two main pathways: the Ubiquitin-proteasome system (UPS) and the Autophagy-lysosome pathway (ALP) (Martin et al., 2023; Aversa et al., 2016). The UPS is responsible for degrading damaged or malfunctioning intracellular proteins. This process begins with tagging the target by adding ubiquitin chains, a process mediated by MuRF1 and atrogin-1, also known as Muscle Atrophy F-box (MAFbx) (Foletta et al., 2011). These ubiquitinated proteins are then recognized and directed to the proteasome, a proteolytic complex that degrades the tagged proteins into smaller peptides (Foletta et al., 2011). In CC, there is increased expression of E3 ligases, what facilitates the ubiquitination of muscle proteins, leading to enhanced protein degradation and consequent muscle mass loss (Martin et al., 2023). Simultaneously, ALP, which generally maintains cellular homeostasis by degrading damaged organelles, is pathologically increased in CC, contributing to muscle atrophy through the degradation of both fiber proteins and organelles (Sandri, 2013). Catabolic factors derived from the tumor, such as proteolysis-inducing factor (PIF), directly activate the UPS, further exacerbating muscle proteolysis (Fearon et al., 2012).

Fearon et al. (2012) state that the impossibility of reversal of the cachectic state by conventional nutritional support is a marking feature of CC. Nevertheless, extensive research has been carried out on the potential of novel nutritional supplements to mitigate the syndrome’s deleterious impact, and understanding the balance between protein synthesis and degradation pathways is mandatory in this scenario. There is no standardized treatment for CC. Various nutritional intervention strategies have shown promise in preventing and treating this syndrome, with some nutrients or bioactive compounds demonstrating beneficial effects, whether alone or in combination. The mechanisms through which these interventions exert their effects on muscle mass are still uncovered, yet they offer a potential therapeutic avenue. One postulated mechanism by which nutrients can mitigate cachexia malnutrition is interfering with systemic inflammation. Overall, this review aims to explore the most studied nutritional supplements and strategies for treating and/or preventing muscle loss in CC.

### 1.1 Protein intake and turnover

Although recognized as the primary anabolic stimulus in skeletal muscle metabolism, studies investigating the effects of increased total protein intake in cancer patients are relatively recent. As revised by Prado and colleagues (Prado et al., 2020), current guidelines for adequate protein intake in oncologic patients fail to address factors such as body composition and muscle depletion. The recommended protein intake for cancer patients ranges from 1 to 1.5 g/kg (Muscaritoli et al., 2021), which appears to be insufficient. Colorectal cancer patients submitted to a high-protein diet at the pre-cachexia stage presented a reduction in subclinical inflammation, one of the main drivers of muscle wasting in cachexia, as well as an improvement in the nutritional status and in appetite (Ziętarska et al., 2017). Also, a systematic review (Capitão et al., 2022) comprising different types of cancer associated with sarcopenia (head and neck, lung, esophageal cancer) reported a minimum amount of protein of 1.4 g/kg to ensure muscle mass maintenance in this population, with lower amounts being associated with muscle loss along the treatment.

It is also important to consider the debate on higher protein intake and its association with tumor growth, since protein synthesis in the tumor, muscle, and immune cells involves the same signaling pathways (Butler et al., 2021; Li et al., 2007). A study with a model of cachectic colon tumor-bearing rats undergoing chemotherapy (Boutière et al., 2023) demonstrated that trends in tumor growth and response to chemotherapy remained unaltered despite enhanced protein dietary intake. Moreover, a modest improvement in nutritional status was observed in animals submitted to a high-protein diet, with an increase in relative fat-free mass. However, in neither of these studies, differences were reported regarding protein metabolism in skeletal muscle.

Most recently, a randomized clinical trial reported the effects of the total daily protein intake on patients with stage II-IV colorectal cancer submitted to chemotherapy (Ford et al., 2024). At baseline, most patients had lower protein ingestion than recommended by the guidelines, and individuals who managed to increase their protein ingestion favored the maintenance of muscle mass, physical function, and anabolism. One of the goals of the study was to assess the response to a diet containing 2.0 g/kg/day of protein versus another, in which 1.0 g/kg/day of protein was consumed, but only 35,3% of the patients in the 2.0 g/kg/day achieved the recommended protein intake. Despite not having succeeded in increasing protein ingestion to the proposed values, the study recognizes that individualized nutritional counselling had a promising effect on protein intake and muscle mass maintenance. Even though CC was an exclusion criterion in this pilot study, it represents the first attempt to demonstrate the effect of nutritional support alone on muscle loss prevention in patients with cancer, as well as providing an evidence-based optimal protein dose for this population, considering features such as acceptance, feasibility and efficacy of the diet regimen.

Along with increasing diet protein intake, the use of specific proteins and amino acids has also been the target of many studies. Among the strategies examined, we chose to address those with clearer evidence of beneficial impact.

### 1.2 Whey protein supplementation

The high concentration of easily digestible essential amino acids in whey protein (WP) renders it ideal as an effective way to add up proteins in the patient’s diet. WP has thus become a suitable choice for providing protein support for cancer patients, as it offers greater nutritional value and faster absorption compared to alternative dietary protein sources (Ramani et al., 2024).

WP is particularly rich in the branched-chain amino acids valine, leucine, and isoleucine, which play a crucial role in tissue growth and repair (Brestenský et al., 2015). Additionally, whey protein is a valuable source of cysteine and methionine, which are glutathione precursors and a key component for enhancing immune function (Mir Khan and Selamoglu, 2020). The whey protein subfractions most studied for their ability to disrupt tumor pathways include α-lactalbumin, bovine serum albumin, and lactoferrin (Teixeira et al., 2019). *In vitro* and *in vivo* research has demonstrated their anticancer properties, including decreased tumor occurrence, growth suppression, and improved antioxidant activity. Furthermore, it can potentially improve conventional cancer treatment efficacy and reduce side effects (Teixeira et al., 2019). In addition, WP has been shown to enhance immune function, improve nutritional status, and promote overall health, making it a beneficial strategy for cachectic patients (Zhao et al., 2022). Nonetheless, changes in muscle metabolism, such as a lower anabolic potential, are common in advanced CC (Prado et al., 2013), where protein synthesis remains impaired even when sufficient protein is consumed. Therefore, nutritional strategies should focus on both the increase of muscle protein synthesis and decrease of anabolic resistance (Van de Worp et al., 2020; Antoun and Raynard, 2018).

In a clinical trial involving cachectic patients undergoing chemotherapy and receiving nutritional supplementation, adding WP to the 3-month treatment regimen significantly improved body composition, muscle strength, and body weight, while reducing chemotherapy toxicity (Cereda et al., 2019).

Several studies indicate that WP supplementation, which is rich in leucine and other essential amino acids, can stimulate protein synthesis more effectively than other protein sources. This effect is attributed to the rapid digestion of WP, which leads to a swift increase in plasma amino acid levels, particularly essential amino acids, potentially mitigating muscle loss in cachectic patients (Cereda et al., 2019; Dangin et al., 2002; Deutz et al., 2011a; Dillon et al., 2007).

Therefore, WP may benefit patients with cancer-associated cachexia by improving muscle synthesis and immune function and reducing inflammation. While WP supplementation shows promise in improving clinical outcomes and quality of life, further studies are needed to define the optimal dosage and evaluate long-term effects.

### 1.3 Branched-chain amino acid (BCAA) supplementation

The amino acids known as BCAA, leucine, isoleucine, and valine, are essential amino acids that play an important role in muscle metabolism. BCAAs have been studied in CC in regard to eventual potential to mitigate muscle mass loss (Ananieva et al., 2016). They interfere with the activation of catabolic pathways, such as the ubiquitin-proteasome system, which are exacerbated in cachexia (Setiawan et al., 2023). BCAA supplementation has been associated with improvement in muscle strength and function, modulation of inflammation, and improvement in energy metabolism, contributing to better quality of life (Storck et al., 2020; Gala et al., 2020).

The benefits of BCAA supplementation, including improved post-chemotherapy recovery - characterized by weight gain and increased energy levels - have been broadly examined (Deutz et al., 2011b; Nojiri et al., 2017; Katagiri et al., 2020; Hachiya et al., 2020). It has also been suggested that BCAAs may increase mitochondrial biogenesis (Valerio et al., 2011), potentially benefiting skeletal muscle energy metabolism (Borack and Volpi, 2016; van Dijk et al., 2015). On the other hand, recent studies have reported that BCAA supplementation may be detrimental to cancer patients, as potentially, tumor cells can consume these amino acids and thus remain alive even within anaerobic environments (Ananieva and Wilkinson, 2018; Ericksen et al., 2019; Lei et al., 2020; Lieu et al., 2020; Peng et al., 2020; Taherizadeh et al., 2021; Tang et al., 2010; Wang et al., 2018). Although limited by the absence of a control group and different types of tumors, two studies showed improvement in patient strength and quality of life (Van der Meij et al., 2019; Zanetti et al., 2020).

BCAAs act as metabolic regulators, influencing not only protein synthesis but also lipid and glucose metabolism (Zhang et al., 2017). Most circulating BCAAs are reincorporated into proteins, functioning as building blocks for muscle synthesis and providing nitrogen for the biosynthesis of nucleotides and nonessential amino acids (Jung et al., 2021). Supplementation in cachectic patients should be carefully monitored, as excessive intake of BCAAs may lead to an imbalance in the availability and metabolism of other essential amino acids (Storck et al., 2020), while the potential effect on tumor progression should be monitored.

#### 1.3.1 Leucine

Leucine is an essential amino acid from the BCAA group. It is known to be an agonist of the mTOR pathway, playing an important role as an anabolic mediator in protein metabolism (Ananieva et al., 2016; Tian et al., 2019). Studies have shown that leucine may play a role in immune function by activating T cells (Ananieva et al., 2016) and regulating the immune response through mTOR signaling, which is crucial in regulating pro- and anti-inflammatory cytokines (Thomson et al., 2009; Soares et al., 2020; Van der Ende et al., 2018).

A leucine-rich diet has shown promising effects in preserving serum insulin concentration along cancer progression, thus promoting muscle protein synthesis and attenuating anabolic resistance without impacting tumor growth in animal models (Cruz et al., 2019). Studies performed by Salomão et al. (2010) and Viana et al. (2016) reinforce these observations, having demonstrated that leucine supplementation can be an effective strategy to improve protein metabolism in cancer patients (Salomão et al., 2010; Viana et al., 2016).

Research in tumor-bearing animals (Cruz et al., 2019; Viana et al., 2016; Gomes-Marcondes et al., 2003; Peters et al., 2011; Xia et al., 2017) and studies with older sarcopenic individuals (Rasmussen et al., 2016; Dickinson et al., 2013) suggest that leucine is a safe and efficient supplement. It positively affects protein metabolism, muscle mass gain, protein and caloric intake, and effectively modulates the inflammatory process.

Although research has shown that leucine can reduce muscle degradation and improve protein metabolism and the inflammatory profile during cancer progression, few studies have evaluated its direct effects upon tumor growth, vascularization, and proliferation. More research is thus needed to understand its impact in the context of human cancer.

#### 1.3.2 β-hydroxy-β-methylbutyrate (HMB) supplementation

HMB is a metabolite derived from leucine metabolism. It has therapeutic potential in CC due to its effects in stimulating protein synthesis and in inhibiting muscle degradation. HMB was initially employed to improve wound healing (Williams et al., 2002), as this metabolite shows significant capacity to enhance muscle protein accretion and to induce collagen renewal (Zanchi et al., 2011).

The anabolic potential of HMB supplementation has been explored for the treatment of muscle loss in cancer, with results pointing out to its capacity to improve long-term outcomes (Prado et al., 2022). Tumor-bearing models show that HMB supplementation attenuates body weight and muscle mass loss at a dose similar to that adopted for human beings. Furthermore, HMB presents antitumor, anti-inflammatory, and anti-cachectic effects (Nunes et al., 2008; Zaira et al., 2011) Other studies showed increased survival time and promotion of favorable metabolic changes (Caperuto et al., 2007) and a significantly larger fiber cross-sectional area after HMB supplementation (Hao et al., 2011). Therefore, HMB supplementation may be a good option for complementary cancer therapy.

A more recent systematic review provided evidence that HMB supplementation benefits muscle mass and function in cancer patients. Additionally, the supplement has been demonstrated to be safe and tolerable, with no adverse effects (Prado et al., 2022). Studies suggest that HMB may effectively mitigate proteolysis associated with cachexia because of its capacity to reduce the activity of the ubiquitin-proteasome system, the main pathway for protein degradation and caspase activity (Prado et al., 2022). Furthermore, HMB stimulates proteogenesis through direct action on the mTOR pathway, the canonical protein synthesis pathway. HMB can also promote the activation of satellite cells in skeletal muscle and potentially enhance the tissue’s regenerative capacity, directly affecting cell proliferation and differentiation (Chodkowska et al., 2018; Yamada et al., 2022).

Nutrients and exercise activate the mTOR pathway in muscle, supporting muscle anabolism. In contrast, in cancer cells, genetic mutations can lead to chronic hyperactivation of the mTOR pathway, fueling tumor growth and bypassing the standard regulatory inhibitory mechanisms. However, studies have not shown a direct link between HMB supplementation and tumor growth (Tian et al., 2019; Prado et al., 2022).

HMB has also been proposed to operate through its capacity to stabilize the sarcolemma via cholesterol synthesis. It has been shown that the majority of HMB is converted into 3-hydroxy-3-methyl-glutaryl-coenzyme A reductase, which is the limiting step of cholesterol synthesis. As such, increased intramuscular levels of HMB may serve as the available substrate for cholesterol synthesis, and therefore for formation and stabilization of sarcolemma (Rossi et al., 2017). HMB is recognized as an effective anti-catabolic agent, reducing protein degradation while enhancing protein synthesis. The currently recommended daily dose is 3 g/day (Muscaritoli et al., 2021).

### 1.4 Glutamine supplementation

Glutamine is the most abundant amino acid in the plasma and in the skeletal muscle (Cruzat et al., 2018). In healthy individuals this amino acid plays an essential role in the regulation of cellular functions, among which immune cell activity, energy metabolism, maintenance of intestinal mucosal integrity, and protein synthesis (Newsholme et al., 2003). Glutamine metabolism is, however, disturbed in cancer patients, resulting in dysregulated protein synthesis, which can contribute to muscle wasting (Cruzat et al., 2018). Thus, the therapeutic potential of glutamine supplementation in cancer patients has been widely discussed, under the light of contributing to the preservation of muscle mass and warranting quality of life in patients with cancer-related wasting (Pradhan et al., 2024).

A cohort of 44 surgery patients with head and neck cancer received enteral glutamine supplementation for 4 weeks, having achieved significantly higher nutritional status and improved clinical outcomes than the control group. Notably, the intervention group maintained lean body mass, which correlated with a higher quality of life score during the postoperative period (Azman et al., 2015). Furthermore, other trials have investigated the effects of glutamine supplementation in combination with HMB and arginine, attenuating cancer-related wasting in association with increased free fat mass in patients with advanced disease, as observed by May et al. (2002).

In animal models, several studies demonstrated improvement in energy balance and inhibition of tumor growth following glutamine supplementation, as reviewed by Van de Worp et al. (2020). Walker-256 tumor-bearing rats supplemented with 2% l-glutamine exhibited reduced body weight loss and a lower percentage (%) loss of body mass index (BMI) (cachexia index), calculated according to tumor mass (Fracaro et al., 2016). These studies suggest that glutamine supplementation may improve muscle preservation and energy metabolism in CC patients.

Besides the promising findings, glutamine supplementation raises concerns due to its role in tumor growth. Since there is metabolic competition for glutamine between host cells and tumor cells, it is crucial to find a balance that supports normal cell function without promoting tumorigenesis during supplementation (Wang et al., 2024; Muranaka et al., 2024). Therefore, glutamine supplementation needs to be carefully monitored, and further research evaluating the potential benefits related to muscle loss in cancer-associated cachexia is necessary.

### 1.5 Arginine supplementation

Arginine is a conditionally essential amino acid that depends on metabolic status. Arginine stimulates cell growth and protein synthesis by inducing the activation of the mTOR pathway in the muscle (Panwar et al., 2023). In addition, arginine serves as a substrate for nitric oxide synthesis, an important signaling molecule that affects immune function, vasodilation, and cicatrization (Wu et al., 2021). Considering its importance in several biological functions, this amino acid has been suggested to improve patient outcome in CC by regulating tumor growth and promoting anabolic effects in the muscle (Pradhan et al., 2024). In addition, in immune system cells, arginine supplementation has been shown to promote beneficial immunomodulatory gain. Finally, it has been shown to improve nutritional status (Soares et al., 2020).

Under catabolic conditions in cancer, arginine levels in plasma are decreased, as observed in patients with breast cancer, colon cancer, and pancreatic cancer, independent of weight loss or tumor stage (Vissers et al., 2005). This result indicates disturbed arginine metabolism in the disease.

A study with arginine supplementation during the perioperative period showed no beneficial effect upon outcome in patients with head and neck cancer; nonetheless, the arginine-supplemented group demonstrated a trend toward improved survival span (Van Bokhorst-de van der Schueren et al., 2001), consistent with findings reported by the same group in a previous study (Buijs et al., 2010). Antoun and Raynard (2018) discussed that arginine supplementation still lacks robust clinical evidence regarding its benefits on muscle wasting. Randomized trials with larger sample sizes, focusing on arginine supplementation in patients with cancer-associated cachexia, may in the future elucidate arginine’s direct and indirect effects on wasting conditions.

### 1.6 Creatine supplementation

Creatine is synthesized endogenously from three amino acids. The initial stages occur in the kidney, where arginine and glycine are involved, and the subsequent steps in the liver, with the participation of methionine. It can also be acquired through an animal protein-rich diet (Tanaka et al., 2022). Most of creatine is absorbed, stored, and used by the skeletal muscle (Jung et al., 2013; Gualano et al., 2012), suggesting creatine supplementation to be able to increase muscle strength and lean body mass in myopathies (Harris et al., 1992).

The effects of creatine supplementation in cachectic patients must still be well established. A study with patients with colorectal cancer under chemotherapy and with CC showed no changes in neither muscle mass, nor body composition (Jatoi et al., 2017). Another randomized and double-blind clinical trial was carried out with 30 individuals with stage III-IV colorectal cancer who received creatine or placebo over an 8-week period. The creatine supplementation protocol included an initial loading phase of 4 × 5 g per day during the first week, followed by a maintenance phase of 2 × 2.5 g per day. Creatine was not effective in improving muscle mass gain or its function. In addition, there were no changes in the quality of life of these patients. However, it enhanced the bioimpedance phase angle, which is related to improved prognosis (Norman et al., 2006).

Studies in rats with cachexia showed that creatine supplementation attenuated weight loss and decreased tumor growth (Cella et al., 2020; Campos-Ferraz et al., 2016; Deminice et al., 2016; Wei et al., 2022). In addition, supplementation promoted lower plasma concentration of TNF-α and IL-6 (pro-inflammatory cytokines), while increasing the concentration of IL-10 (anti-inflammatory cytokine), and preventing atrogin-1 and MuRF-1, key regulators of muscle atrophy in the skeletal muscle (Cella et al., 2020).

Although the recommended dose for humans is 3–5 g/day (Tanaka et al., 2022), this dose did not show the same results in patients with cachexia (Jatoi et al., 2017; Norman et al., 2006), suggesting that changes in the metabolic pathways associated with the disease may limit the effects of creatine (Tanaka et al., 2022).

### 1.7 L-carnitine supplementation

L-carnitine (LC) is among the most studied nutritional supplements in advanced cancer patients with malnutrition (Johal et al., 2022) due to its antioxidant activity and possible anti-wasting effect (Alhasaniah, 2023). It is synthesized in the liver and kidney by converting the amino acids lysine and methionine. Its primary function is to facilitate the transport of long-chain fatty acids to the mitochondrial matrix for β-oxidation and subsequent energy production (Alhasaniah, 2023; Longo et al., 2016). Failure in this process can lead to increased oxidative stress, metabolic dysfunctions, and increased pro-inflammatory cytokines. In this context, LC supplementation can reduce oxidative stress and the inflammatory response, leading to potential clinical benefits (Longo et al., 2016).

Gramignano et al. (2006) evaluated the effect of LC supplement intake in 12 patients with advanced cancer, having observed a significant increase in lean mass and an improvement in appetite after 4 weeks, suggesting that LC can mitigate muscle loss and improve nutritional status. In a phase III study by Mantovani et al. (2010), although the use of a LC supplement (4 g/day) for 4 months did not significantly improve primary outcomes, such as lean mass and fatigue, a positive impact on secondary indicators such as the Glasgow Prognostic Score and ECOG (Eastern Cooperative Oncology Group) performance status was reported. Similarly, the study by Kraft et al. (2012) in patients with advanced pancreatic cancer showed that LC supplementation (4 g/day) for 12 weeks increased BMI and improvement in quality of life, further supporting the therapeutic potential of LC in patients with cancer.

In experimental models, LC treatment improved food intake, reduced muscle mass loss, and led to the downregulation of atrogin-1 and MuRF1, biomarkers of skeletal muscle atrophy. In addition, LC intake decreased proteasome activity (degradation pathway) in the gastrocnemius muscle of cachectic animals (Busquets et al., 2012; Busquets et al., 2020) and increased physical activity levels by improving energy production (Busquets et al., 2012). Another intervention with the LC supplement led to an increase in both food intake and muscle mass. The study also examined the activity of carnitine palmitoyltransferase-1 (CPT-1), a marker of the effects of carnitine. The results demonstrated an upregulation of CPT-1, associated with reduced plasma levels of IL-6 and TNF-α (Liu et al., 2011). Other experimental studies support the finding that CPT-1 activity is higher in the muscles of cachectic animals supplemented with LC (Busquets et al., 2020; Silvério et al., 2012) in relation to controls. In cachectic animals, CPT-1 expression is typically reduced, contributing to an accumulation of triacylglycerols in the liver. A 28-day supplementation of LC administered intragastrically at a dose of 1 g/kg body weight/day, effectively restored CPT-1 activity, thereby preserving hepatic lipid metabolism. Additionally, LC modulated CPT I enzymatic activity and MTP gene expression, further supporting healthy hepatic lipid regulation. The supplementation also enabled normal weight gain in tumor-bearing animals and significantly inhibited tumor growth, demonstrating LC’s potential as a therapeutic strategy for managing cachexia (Silvério et al., 2012).

The available evidence suggests that LC has the potential to mitigate muscle mass loss, modulate lipid metabolism, and reduce inflammation associated with CC. Nonetheless, further studies are required to investigate the underlying molecular mechanisms to understand its therapeutic effects better.

### 1.8 Omega-3 fatty acids supplementation

Marine long-chain omega-3 (n-3) fatty acids, eicosapentaenoic acid (EPA) and docosahexaenoic acid (DHA) are well-known for their anti-inflammatory properties. N-3 has been shown to decrease tumor angiogenesis and invasiveness (McCarty, 1996). N-3 supplementation can alter inflammatory markers in patients with colorectal cancer, decreasing IL-6 and increasing albuminemia (Camargo et al., 2016). Additionally, n-3 polyunsaturated fatty acids can induce ferroptosis through lipid peroxidation in acidic tumor environments, selectively promoting cancer cell death (Dierge et al., 2021), while also inhibiting tumor proliferation and modulating pro-resolving lipid mediators, which help suppress chronic inflammation (Liput et al., 2021).

We have previously discussed in a meta-analysis that n-3 fatty supplementation was not able to lower circulating inflammatory markers in cachexia (de Castro et al., 2022). Nevertheless, due to the low number of studies that matched our inclusion and exclusion criteria, only six articles were included in the systematic review, disallowing a strong conclusion (de Castro et al., 2022). Oral nutritional supplements with high protein and n-3 content have been shown to preserve lean body mass during chemo (radio) therapy (de van der Schueren et al., 2018). However, another meta-analysis could not support this result, indicating that oral n-3 supplements could not maintain muscle and body weight mass nor improve the quality of life in patients with cancer (Lam et al., 2021).

Despite these contradictions, the European Society for Clinical Nutrition and Metabolism (ESPEN) advocates, although with a weak recommendation, the supplementation of n-3 for some patients with cancer (Muscaritoli et al., 2021). Notably, patients with advanced cancer undergoing chemotherapy and endangered by weight loss or malnutrition could benefit from n-3 supplementation due to its ability to improve appetite, food consumption, lean body mass, and body weight (Muscaritoli et al., 2021). The safety and tolerability of n-3 supplements were also highlighted (Muscaritoli et al., 2021).

### 1.9 Antioxidants

An increase in oxidative stress occurs when the normal redox equilibrium is altered, with increased presence of oxidative species and lower antioxidant defense status (Li et al., 2022). Patients with lung cancer and cachexia have higher skeletal muscle protein carbonyls and superoxide anions content compared with healthy controls (Puig-Vilanova et al., 2015). It has been proposed that dietary antioxidants may modulate the oxidative stress in CC (Li et al., 2022).

A comprehensive meta-analysis including approximately 68 clinical trials did not identify protective effects of antioxidants against cancer. On the contrary, an increase in mortality was observed in individuals who consumed β-carotene, vitamin A or vitamin E, as evidenced by Bjelakovic et al. (2007). These findings can be partially explained by the critical role of free radicals as signaling molecules in anabolic processes. Excess antioxidants can negatively interfere with these signaling pathways, compromising essential cellular mechanisms (Higgins et al., 2020).

Reactive oxygen species (ROS) play a complex role in cancer biology. Elevated ROS levels are essential for tumor cell proliferation, survival, and progression by activating signaling pathways such as NF-κB, STAT3, and MAPK, which promote cell growth, angiogenesis, and metastasis. Paradoxically, ROS are critical for the efficacy of chemotherapy, as many anticancer treatments rely on elevating ROS to toxic levels to induce oxidative stress and trigger tumor cell death. Excessive antioxidant supplementation can disrupt this delicate balance by lowering ROS levels below the threshold necessary for effective chemotherapeutic action (Moloney and Cotter, 2018). This highlights the complex role of ROS and the potential risks of antioxidant overuse in compromising tumor control and treatment outcomes.

#### 1.9.1 Resveratrol (trans-3,4′,5-trihydroxystilbene) supplementation

Resveratrol is a polyphenol in the skin of red grapes and other fruits. It has been shown to inhibit NF-κB (p65) activity and gene expression of MURF1 in the skeletal muscle of mice bearing C-26 adenocarcinoma (Shadfar et al., 2011). In mice with lung cancer and cachexia, resveratrol (20 mg/kg body weight/day, for 15 days) was able to reduce tumor mass and preserve body weight, soleus and gastrocnemius weight, myofiber cross-sectional area, and decrease Atrogin and MURF1- protein (Penedo-Vázquez et al., 2021). However, in a previous study, resveratrol administered to rats bearing the Yoshida AH-130 ascites hepatoma (1 mg/kg body weight/day for 7 days) and mice bearing the Lewis lung carcinoma (at doses of 5 and 25 mg/kg body weight/day for 15 days) was not able to preserve skeletal muscle and body weight mass (Busquets et al., 2007). Of concern, rats bearing the Yoshida AH-130 ascites hepatoma that received resveratrol showed lower food intake, lower gastrocnemius mass, heart mass, and white adipose tissue and liver weight, compared with rats bearing the tumor that did not receive resveratrol (Busquets et al., 2007).

#### 1.9.2 Curcumin supplementation

Curcumin is a polyphenolic compound found in turmeric. It is recognized as a potent antioxidant and has been studied in the context of CC (Li et al., 2022). Patients with pancreatic cancer who received 8 g of curcumin per day for up to 18 months showed lower expression of NF-κB proteins, cyclooxygenase-2, and phosphorylated signal transducer and activator of transcription 3 in peripheral blood mononuclear cells (Dhillon et al., 2008). In a lung cancer-induced cachexia model, treatment with curcumin (1 mg/kg body weight/day for 15 days) effectively mitigated muscle wasting. Curcumin effectively preserved body weight and increased the weight of both the gastrocnemius and soleus muscles. Additionally, it enhanced the cross-sectional area of type I and type II muscle fibers, indicating reduced muscle atrophy. Furthermore, it attenuated proteolysis by reducing the levels of Atrogin-1 and MuRF-1, while also improving overall muscle structure and function (Penedo-Vázquez et al., 2021). Patients with advanced colorectal cancer received doses between 0.45 and 3.6 g per day for up to 4 months (Sharma et al., 2004). A dose of 3.6 g per day decreased the production of a lipid mediator derived from arachidonic acid, prostaglandin E2 (PGE2), in blood samples taken 1 h after the dose on days 1 and 29, compared to the PGE2 concentration found before treatment (Sharma et al., 2004).

#### 1.9.3 Epigallocatechin-3-gallate supplementation

Epigallocatechin-3-gallate (EGCG), a polyphenol derived from green tea, shows promising properties in the context of cancer and cachexia. Studies have shown that EGCG negatively regulates the expression of genes associated with Atrogin-1, a key protein in the ubiquitin-mediated protein degradation process, while also acting on other proteins of the F-BOX family, contributing to the prevention or mitigation of the progression of cachexia both *in vitro* and *in vivo* (Wang et al., 2011). Additionally, EGCG exhibits anti-inflammatory properties, which may alleviate the systemic inflammation characteristic of cancer-associated cachexia, thus reducing the overall metabolic burden of the organism (Loyala et al., 2024).

Furthermore, EGCG inhibits tumor growth by inducing apoptosis through the mitochondrial pathway, arresting the cell cycle, and modulating key signaling pathways such as EGFR/RAS/RAF/MEK/ERK. These combined effects highlight its potential as a therapeutic agent against both cancer progression and muscle wasting (Sharifi-Rad et al., 2020).

A randomized, placebo-controlled trial assessed the safety of green tea catechins (Polyphenon E^®^) in 97 men with high-grade prostatic intraepithelial neoplasia (HGPIN) or atypical small acinar proliferation (ASAP) over 1 year. Participants received 200 mg of EGCG twice daily with food. The supplement was well-tolerated, with no significant treatment-related adverse events, including liver toxicity, compared to placebo. Plasma EGCG levels were significantly higher in the treatment group, confirming compliance (Kumar et al., 2016). The same group also showed that the supplemented group had lower rates of prostate cancer and Gleason scores after 1 year. Notably, in men with HGPIN but not ASAP at baseline, no progression to ASAP occurred in the treatment group compared to 20% in the placebo group (Kumar et al., 2016) These findings highlight the potential of green tea catechins to slow the progression of precursor lesions without increasing the risk of high-grade disease.

Despite these potential benefits, the mechanisms of action of EGCG are not yet fully elucidated. Green tea catechins exhibit very low stability after digestion, with EGCG being particularly sensitive. Its poor bioavailability restricts its therapeutic potential; however, it can be amplified when combined with other compounds, such as vitamin C, which has been shown to boost its biological activity in experimental models (Furniturewalla and Barve, 2022).

### 1.10 Vitamin D supplementation

Patients with metastatic prostate cancer who received 2000 units of vitamin D per day for 12 weeks showed an improvement in muscle strength. Among the 16 patients enrolled, half of them showed an improvement in the timed chair rises, and the other half improved timed 10-meter walk (Van Veldhuizen et al., 2000). A meta-analysis evaluated the vitamin D levels and the outcomes in patients with lung cancer (Feng et al., 2017). This study showed that circulating 25-hydroxyvitamin D concentration was inversely associated with lung cancer risk and mortality but not with overall survival (Feng et al., 2017).

Higher (4000 IU) compared to standard dose (400 IU) of vitamin D supplementation together with chemotherapy to treat head and neck cancer was not able to improve patients’ body weight, BMI, muscle area, muscle attenuation, visceral adipose tissue area, or subcutaneous adipose tissue area after the first eight cycles of chemotherapy (Brown et al., 2020).

### 1.11 Combined nutrient supplementation

Cancer patients received arginine associated with omega-3 fatty acids and dietary nucleotides 5 days before and after radical cystectomy. They were compared with the control group that received BOOST Plus^®^, a commercially available nutritional drink (Hamilton-Reeves et al., 2018). The supplementation increased arginine levels from baseline to day 30 postoperative and immune support by maintaining the Th1-Th2 balance during surgery, leading to lower plasma IL-6 levels. Despite the advantageous modulation of the inflammatory response, there were no significant differences in appendicular muscle loss between the two groups (Hamilton-Reeves et al., 2018).

Chitapanarux et al. (2020) investigated the effects of arginine, glutamine, and fish oil supplementation in 88 cancer patients with head and neck cancer (45%), esophageal cancer (32%), and cervical cancer (23%) undergoing concurrent chemoradiotherapy (CCRT). The intervention group received 250 mL of supplementation twice daily, providing 500 kcal/day with additional protein (106.25 g/day total) for 40 days on average, corresponding to the full course of CCRT. The intervention group showed significantly lower severe hematologic toxicities (5% vs 23%, p = 0.03) and improved treatment completion rates compared to the placebo group. The study highlights the potential of immune-enhancing supplements to reduce CCRT-related toxicities and improve treatment adherence. Nonetheless, it may not be the exclusive beneficial factor, as it was not an isocaloric study, and the supplemented group received additional protein and calories (500 kcal/d) from the arginine, glutamine, and fish oil supplementation.

May et al. (2002) conducted a randomized, double-blind, controlled study to assess the effects of HMB, arginine, and glutamine supplementation in patients with advanced cancer cachexia. Thirty-two patients with solid tumors received either the HMB/Arg/Gln mixture (3 g HMB, 14 g arginine, 14 g glutamine daily) or an isonitrogenous control for 24 weeks. The supplemented group experienced an increase in body weight, primarily due to gains in lean body mass, while the control group showed continued muscle loss. These improvements were maintained throughout the study, and body composition changes were validated through multiple assessment methods. The supplementation was well-tolerated, with no adverse effects reported. Although no significant changes in fat mass or quality of life were observed, the results suggest that HMB, arginine, and glutamine may help counteract muscle wasting in CC, possibly by reducing protein breakdown and enhancing protein synthesis (May et al., 2002).

Changes in muscle mass and function were evaluated in 55 pre-cachectic and cachectic patients with lung cancer, who received an oral nutritional supplement containing ≈200 kcal, 10 g whey protein, 2.0 g eicosapentaenoic acid/docosahexaenoic acid in fish oil, and 10 µg 25-hydroxy-vitamin D3 during a 12-week randomized, double-blind, controlled pilot trial. The group of patients that received the supplement did not show differences in waist and calf circumference, appendicular lean body mass, grip strength, or daily walking distance compared to the control group (Laviano et al., 2020).

Another study evaluated the effects of an oral nutritional supplement containing several compounds, including vitamin C, vitamin B5, vitamin B9, and vitamin D alongside nutritional counseling, offered to 30 patients with lung cancer under chemotherapy (Torricelli et al., 2020). Compared to the control group, the patients who received the nutritional supplement maintained or increased their body weight after 90 days and reported improvements in chemotherapy-related symptoms, including anorexia, weakness, dyspnea, pain, and hemoptysis, leading to enhanced quality of life (Torricelli et al., 2020).

## 2 Conclusion

**FIGURE 1:**
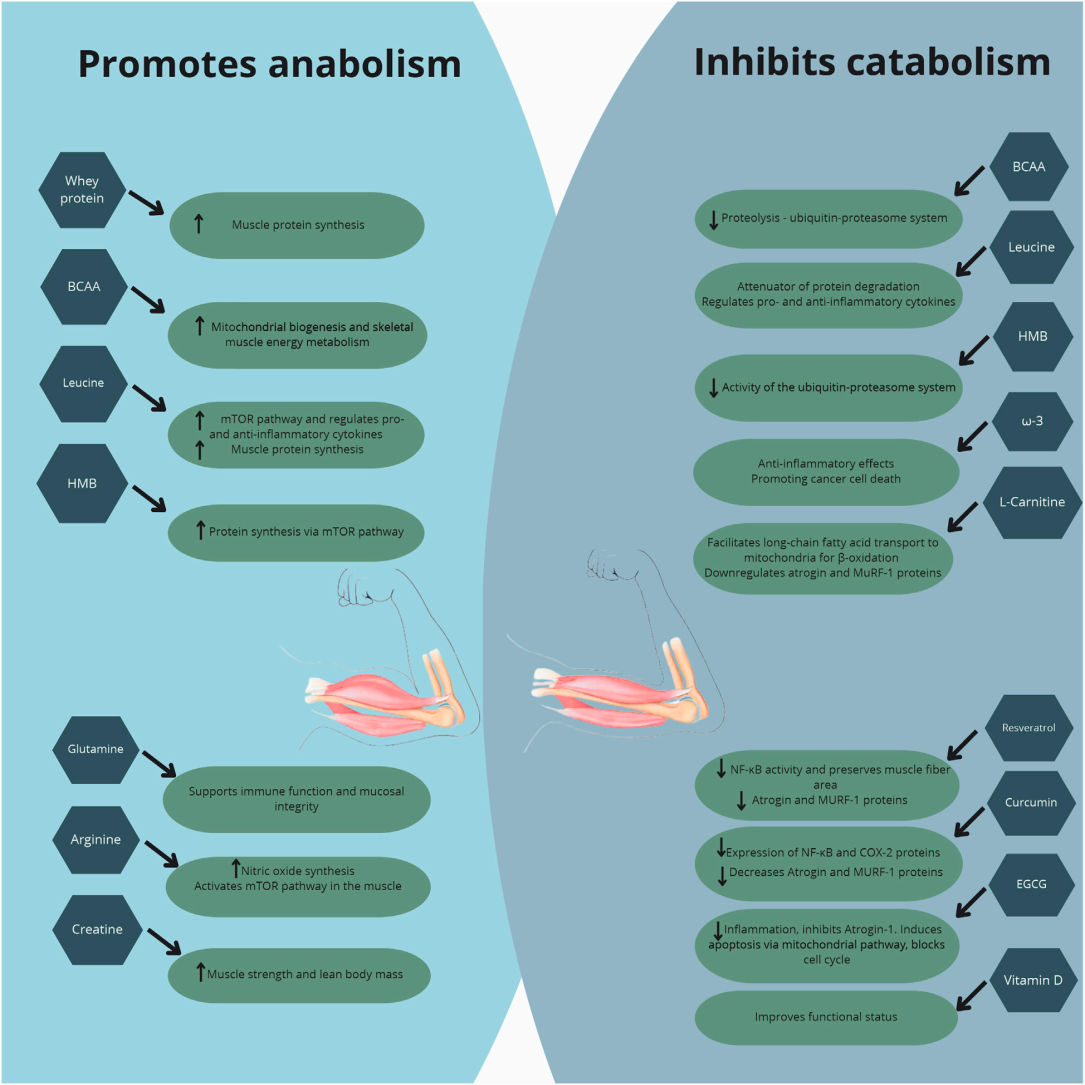
Major effects of nutritional supplementation in cancer cachexia.

Nutritional intervention strategies have been explored owing to the potential impact on mitigation of muscle loss in patients with CC (Figure 1), a multifactorial condition marked by accelerated muscle protein degradation. Most cancer patients face treatment and disease side effects, compromising food intake of calories, protein, and bioactive compounds, worsening the nutritional status and aggravating the inflammatory and catabolic state.

To maximize the anabolic potential, it is crucial to detect risks of significant weight loss at the time of diagnosis and, through continuous nutritional screening, provide early nutritional intervention to prevent compromising of clinical outcome.

High-protein diets were reported to be associated with maintenance of muscle mass, while WP supplementation, a good strategy to increase protein intake, stands out for allowing rapid absorption and high plasma concentration of essential amino acids. WP improved body composition, muscle strength, and overall treatment tolerance in cancer patients undergoing chemotherapy. Additionally, HMB emerges as a promising option due to its dual action of promoting protein synthesis and inhibiting muscle degradation.

Creatine supplementation has yielded inconsistent results in human studies, with no significant improvements in muscle mass or quality of life observed in colorectal cancer patients. Despite promising results in improving lean mass and reducing fatigue in advanced cancer patients, LC requires further investigation to confirm efficacy.

The use of omega-3 fatty acids has shown contradictory results. When effectiveness was evaluated by meta-analysis, supplementation had no significant impact on inflammatory markers or muscle mass maintenance. Similarly, while antioxidants like resveratrol, curcumin, and EGCG have been explored for potential benefits, the clinical evidence in humans remained limited and inconclusive.

Combined nutrient supplementation, including arginine, omega-3 fatty acids, glutamine, and HMB, has demonstrated varying degrees of benefit in cancer patients by modulating the inflammatory response, reducing treatment-related toxicities, and preserving lean body mass. While improvements in immune function, symptom control, and quality of life were observed, the impact on muscle preservation and physical function remains inconsistent across studies.

In conclusion, the management of cancer-associated cachexia requires an individualized approach associated with nutritional counseling to manage treatment and disease side effects and to ensure feasible ways to achieve dietary requirements. An early intervention is mandatory or, at least, as soon as possible. The total protein intake matters and a dose of 1.4–2 g/kg of body weight is more effective in preventing muscle loss. When combined with other nutrients, it may improve the inflammatory profile. Further research, particularly well-designed clinical trials, is needed to determine optimal dosages and evaluate long-term outcomes, especially across different stages of cachexia and in conjunction with other therapeutic approaches to establish their full potential and clinical utility.
